# An assessment of self-reported physical activity instruments in young people for population surveillance: Project ALPHA

**DOI:** 10.1186/1479-5868-8-1

**Published:** 2011-01-02

**Authors:** Stuart JH Biddle, Trish Gorely, Natalie Pearson, Fiona C Bull

**Affiliations:** 1 British Heart Foundation National Centre for Physical Activity & Health, School of Sport, Exercise & Health Sciences, Loughborough University, UK; 2 Institute of Youth Sport, School of Sport, Exercise & Health Sciences, Loughborough University, UK; 3 Centre for Physical Activity and Nutrition Research, School of Exercise and Nutrition Sciences, Deakin University, Australia; 4 School of Population Health, The University of Western Australia, Australia

## Abstract

**Background:**

The assessment of physical activity is an essential part of understanding patterns and influences of behaviour, designing interventions, and undertaking population surveillance and monitoring, but it is particularly problematic when using self-report instruments with young people. This study reviewed available self-report physical activity instruments developed for use with children and adolescents to assess their suitability and feasibility for use in population surveillance systems, particularly in Europe.

**Methods:**

Systematic searches and review, supplemented by expert panel assessment.

**Results:**

Papers (n = 437) were assessed as potentially relevant; 89 physical activity measures were identified with 20 activity-based measures receiving detailed assessment. Three received support from the majority of the expert group: Physical Activity Questionnaire for Children/Adolescents (PAQ-C/PAQ-A), Youth Risk Behaviour Surveillance Survey (YRBS), and the Teen Health Survey.

**Conclusions:**

Population surveillance of youth physical activity is strongly recommended and those involved in developing and undertaking this task should consider the three identified shortlisted instruments and evaluate their appropriateness for application within their national context. Further development and testing of measures suitable for population surveillance with young people is required.

## Background

Physical activity in young people has become a major issue in public health as evidence emerges on the important role of physical activity in many health conditions, including overweight and obesity, type 2 diabetes, cardiovascular disease risk, skeletal health, and mental health. In particular, the issue of obesity in youth, and the link between this condition and type 2 diabetes, as well as the increases in diabetes [1] is topical and currently demanding much attention in physical activity research [2].

Establishing links between physical activity and health outcomes is a fundamental phase of the behavioural epidemiology framework proposed by Sallis and Owen [3]. An early phase of this framework is to identify valid and reliable ways to assess physical activity. If suitable assessment methods can be developed, and health outcomes identified that are associated with physical activity, this logically leads to research identifying factors associated with physical activity ('correlates') and interventions to increase physical activity. However, much of this is predicated on the use of suitable tools for assessing levels of physical activity.

In addition to assessing physical activity in research studies, where researchers may have the opportunity of conducting lengthy and detailed assessments, tools are also needed for assessing population level prevalence [4]. This is the focus of the present paper. Usually such instruments have to be brief as they may sit alongside other health assessments within population surveillance systems [5].

### Assessment Using Self-Report

Until the development of movement sensors, such as pedometers and accelerometers, the assessment method of choice for physical activity has been self-report. Consequently, there are a large number of instruments, with varying degrees of formatting and development, with many aimed at assessment in young people. However, this approach to measurement is fraught with difficulties and consequently there are many poorly developed instruments alongside those that have a history of more robust design and development.

There are many good sources of discussion concerning issues in self-reported assessment of physical activity, in both youth and adults (e.g., [6-10]). Moreover, there are many methods that can be used, including questionnaires/surveys and diaries. Survey methods can require self or proxy completion, the latter, for example, by parents for young children.

In the analysis of self report surveys that follows, we focused on instruments that might be suitable for assessment in large-scale population surveillance, usually for the purpose of estimating the prevalence of physical activity. Prevalence estimates refer to the proportion of the population estimated within physical activity time-based categories (e.g., percentage meeting national guidelines, such as 60 minutes of MVPA each day of the week). This contrasts with possible outputs such as mean estimates of time spent in specific types of physical activity behaviours, or total physical activity behaviour, or 'dose'.

Such assessments are usually part of a wider public health surveillance system. The USA Centres for Disease Control [11] stated that "Public health surveillance systems should be evaluated periodically, and the evaluation should include recommendations for improving quality, efficiency, and usefulness." This will include an evaluation of the type of assessment (e.g., objective or self-report), and continued assessment of the nature of the instrument being used. To this end, Project ALPHA was established, with one aim being to provide guidance on the measurement of physical activity in young people using self-report instruments and assess their suitability for population surveillance monitoring, particularly in the European context (see http://sites.google.com/site/alphaprojectphysicalactivity/). That is, instruments relevant for populations of these types of countries.

In evaluating, as well as developing, self-report instruments for surveillance of physical activity in youth, consideration needs to be given to several key issues [12], including:

•What domains of physical activity are being assessed? These could be general (total physical activity) or in specific contexts, such as school or leisure-time.

•Does the instrument assess the frequency, intensity, duration and type of activity?

•Does the instrument assess the temporal dimension of physical activity (e.g., activity at different times of the day)?

•Over what period are participants being asked to recall their activity?

•Is the instrument suitable for the age group it is aimed at? This will require the testing of items for appropriateness of the language used and consideration of the cognitive capacities of the child being assessed.

•Is the instrument appropriate in respect of ease of completion and participant burden, given that large samples will be required to be tested for population surveillance and prevalence?

•Is the instrument suitably valid and reliable?

This paper addresses the assessment of physical activity for children and adolescents for population level surveillance using self-report instruments. With technological improvements and reductions in costs, population surveillance may, in future, routinely use more objective instruments, such as accelerometers. However, even if this is the case, concomitant assessment of some aspects of physical activity (e.g., type) will require self-report assessment, alongside objective methods.

The purpose of this paper, therefore, is to review existing self-report instruments purporting to assess physical activity in young people. Instruments will be appraised and a short list of measures that may be suitable for population surveillance, with the ability to provide suitable prevalence estimates, will be considered with the purpose of making recommendations on usage, particularly in a European context.

## Method

### Search Strategy

Literature searches, using tailored search terms appropriate to each database, were conducted using Web of Science, Medline, PubMed, PsycINFO, SportDISCUS, and SIGLE. Typical search terms included physical activity, exercise, sport, children, adolescent, boys, girls, infants, measurement, surveillance, and survey. Search terms followed the same order of (1) behaviour terms, (2) population terms, and (3) method terms. The census date for searches was June 2008. We also searched the reference lists of key texts and reviews published since 2000 (e.g., [13-19]), plus the special issues on physical activity measurement in *Medicine and Science in Sports and Exercise *(1997, 29 (6) Supplement; 2000, 32 (2) Supplement) and *Research Quarterly for Exercise and Sport *(2000, 71 (2)).

We also contacted 42 people across 37 European countries asking them to identify any population based survey instruments that were used to measure physical activity in young people within their country. Ten people replied but no new instruments were identified through this process.

Inclusion criteria for instruments to be considered for further evaluation required that studies a) reported the use of a self-report measure of physical activity and b) targeted participants less than 19 years of age. Studies only assessing physical fitness or employing only objective measures were excluded.

The search strategy resulted in 24,190 titles which were initially screened for duplicates and potential relevance. After this initial screening, 1839 titles and abstracts were assessed against the inclusion/exclusion criteria. In total 437 papers were assessed as potentially relevant and full papers were obtained. From these a total of 89 physical activity measures were identified. The measures were grouped as activity-based instruments (n = 67), time-based instruments (n = 5), proxy instruments (n = 17) and observation instruments (n = 1). Activity-based instruments are structured around a list of activities with minimal reference to time of day. By contrast, time-based instruments divide the day into time blocks and respondents provide the dominant activity/energy expenditure for each time block. Proxy instruments are completed on behalf of the individual (often by a parent) and observational tools are used to record behaviour by an observer.

### Data Synthesis

Information about each instrument was extracted and tabulated, including instrument name, indicative references, details of physical activity assessment (including physical activity dimensions assessed, recall period, method and structure of the instrument, number of items, other relevant information, and measurement format), age of participants, indicative countries in which it has been used, and information on reliability and validity. From the initial long table a short list of 20 activity-based instruments and 2 time-based instruments was constructed (Additional file 1, Table S1). Observational and proxy measures were not considered appropriate tools for population surveillance. The inclusion criterion for short-listing was that some level of reliability and/or validity had been demonstrated. After further consideration, based mainly on participant burden and the necessity for the instruments to be suitable for population level surveillance, the two time-based instruments were dropped from further analysis.

The short-listed instruments were summarised within a further table that included instrument name, a rating of validity and reliability, a summary of physical activity assessment (e.g., time frame, number of items, time to complete), age range, use since 1998 (Y/N), previous use in large scale survey (Y/N), availability of European data (Y/N), and comments/qualifications (see Table 1).

**Table 1:** Details of shortlisted instruments, including ratings of validity and reliability

Instrument	Validity demonstrated^1^	Reliability demonstrated^2^	Feasibility					Age range tested	Data since 1998?	Large scale survey data?	European data?
	**(quality of study score/statistical strength)**		**PA domain**	**Time frame**	**# items**	**Admin^3^**	**Time**				

Self-Administered Physical Activity Checklist (SAPAC)	2/2	2	# activities, MVPA minutes, PA MET score	Previous day	21 item checklist	SR	?	11 y +	✔	✘	Some

Adolescent Physical Activity Recall Questionnaire (A-PARQ)	3/1	2	Organised sport, non-organised sport	Normal week	Free response with example activities listed	SR	20 mins	13 & 15 y	✔	✘	✘

Swedish Adolescent Physical Activity Questionnaire (SwAPAQ)	2/2	0	School, transport and leisure time PA, sitting time	Last 7 d	25	SR	?	16-17 y	✔	✘	✔

Physical Activity Questionnaire for Older Children (PAQ-C) Physical Activity Questionnaire for Adolescents (PAQ-A)	3/2	2	MVPA, longer-than-10mins-MVPA	7 d	9	SR	20 mins	8-13 y PAQ-C; 13-20 y PAQ-A	✔	✘	✘

Youth Risk Behaviour Surveillance Survey (YRBS)	3/2	2	School and leisure time PA, strength exercises, sport	Past week or year	5	SR	?	10-21 y	✔	✔	✘

Health Behaviour in School Aged Children (HBSC)	3/1/2	3	Out of school MVPA	Usual frequency & duration	2	SR	?	11-16 y	✔	✔	✔

Children's Leisure Activities Study Survey (CLASS)	2/1	1	Moderate, vigorous, & total PA	Typical week	Check-list	SR PP	?	5-12 y	✔	✘	✘

Godin Leisure-Time Exercise Questionnaire (LTEQ)	3/1	2	Strenuousmoderate and mild exercise for more than 15 minutes	7 d	4	SR (interview lead)	<5 mins	9 y +	✔	✘	✔

Seven-Day Physical Activity Recall (7D-PAR)	3/2	3	Very hard & mod PA for more than 15 mins	7 d		IA	?	11-16 y	✘	✘	✔

Teen Health Survey	3/2	2	MVPA (>= 3 METS)	7 d or typical week	2	SR	?	14-17 y	✔	✔	✘

Four by One-day Recall Physical Activity Questionnaire (4BY1DPAR)	2/2/3	1	PA at school, sport at school, leisure time PA, leisure time sport	24 h × 4 d		SR & IA	?	7-18 y	✔	✔	✔

Computerised Activity Recall (CAR)	2/2	2	All PA and SB of at least 15 min duration	Previous day	~200	SR via computer driven menu	?	10-13 y	✘	✘	✘

Children's Physical Activity Interview	3/2	0	All PA during waking hours	Previous day	Free response directed by interviewer 5 time periods	IA	18 mins	8-11 y	✘	✘	✘

Cardio-vascular Risk in Young Finns	2/2	1	Leisure time PA, sports club/competitions	Habitual	5	SR	?	3-18 y	✔	✔	✔

ACTIVITY video questionnaire	2/2	0	School day PA	1 d	10	SR (by video)	?	7-8 y	✔	✘	✘

Youth Media Campaign Longitudinal Survey (YMCLS)	3/2	2	Out-of-school activities (organised), leisure time PA	1 d & 7 d	Free response	IA (phone)	?	9-13 y	✔	✘	✘

School Health Action, Planning and Evaluation System (SHAPES) Physical Activity Questionnaire	3/3	2	All PA	7 d	45	SR	?	11-17 y	✔	✔	✘

Finnish Twin Cohort Study	2/3	3	Leisure time PA,	Habitual	2	SR	?	16-18 y	✔	✔	✔

Physical Activity and Lifestyle Questionnaire (PALQ)	3/2	3	Leisure time PA	7 d and habitual	27	SR	?	~13 y	✔	✘	✔

Physical Activity and Exercise Questionnaire (PAEQ)	2/1	0	Usual PA	Habitual	3 section s (multiple questions)	SR	?	10-15 y	✔	✔	✘

^1 ^Validity (quality of study score): 0 = none; 1 = weak; 2 = moderate; 3 = strong. Validity (statistical strength): 1 = poor; 2 = fair; 3 = good.

^2 ^Reliability: 0 = none; 1 = weak; 2 = moderate; 3 = strong.

^3 ^SR: self-report; PP: parent proxy report; IA: interviewer administered

Validity and reliability of each instrument was assessed by two of the researchers using criteria shown in Table 2. The absolute value of the statistic reported was assessed first, followed by any other criteria used.

**Table 2:** Table used to guide ratings of instruments

Criteria	Score = 0	= 1	= 2
N	<15	15-49	N > 50

Sample	convenience	narrow	diverse

Validity: Criterion measure		indirect	direct

Reliability: Test-retest period	Same day	1 week	>= 2 weeks

Absolute value of statistic (b)	poor	fair	Excellent/strong

Notes:

a). Validity/Reliability rating:

0 = none

1 = weak (statistical value = 0 or scores 3 or less in other categories)

2 = moderate (statistical value = 1 or 2 and scores 4 or 5 in other categories)

3 = strong (statistical value = 1 or 2 and scores 6 or 7 in other categories).

b). Absolute value of statistics reported: (i) ICC: poor agreement (<.40), fair to good agreement (0.40-0.75), excellent agreement beyond chance (>.75); (ii). r or r_s_: 0.3 fair, 0.5 good/moderate, 0.7 strong; (iii). kappa: poor agreement (<.40), fair to good agreement (0.40-0.75), excellent agreement beyond chance (>.75).

### Expert Assessment

Five international experts in the area of physical activity measurement were invited to contribute to the review by scrutinising a table containing 89 instruments, and provide critical feedback on the short list of 20 instruments. A briefing and discussion took place face-to-face with all experts together. At the meeting, criteria for electronic searches and the assessment of reliability and validity were outlined and discussed. The experts then provided feedback some weeks later after further scrutiny away from the meeting. Specifically, the experts were asked to identify the instruments in the short list table most appropriate for use in population surveillance, with additional comments if necessary. Specifically, they were requested to specify if the instrument was in their 'top 5', and then provide reasons for their decision, including whether they had personal experience of using the scale or whether additional issues needed to be noted (e.g., better for some ages than others; ease of administration) (see Table 3). Finally, they were requested to specify if any other instrument might be missing from the short list. Key papers for each instrument were made available online. Four of the five experts were able to respond by the deadline.

**Table 3:** Feedback from the expert group evaluation and ranking exercise (✔ indicates that instrument was ranked in top 5 by an expert). Only instruments with at least one expect proving support (✔) are included.

Instrument	Top 5?	Reasons	Additional comments (e.g. personal experience, groups to use with)
Adolescent Physical Activity Recall Questionnaire (A-PARQ)	✔	Indirect validity only; moderate reliability. Would require more reliability and validity testing before generalizing to other ages. Instrument looks difficult for older children to understand and coding looks difficult to interpret.	Limited age range 13 & 15 y tested. Easy to administer.

Physical Activity Questionnaire for Older Children (PAQ-C) Physical Activity Questionnaire for Adolescents (PAQ-A)	**✔✔✔**	Consistent high validity against a variety of direct measures including doubly labelled water; only moderate reliability. Feasible, but may be time consuming. 1st choice given European data. Simple to complete, well used, and time efficient.	Pair of instruments tested on widest age range (8-20 y). Continuous measure of pa (MVPA > 10 min). Reasonable recall 7d. Feasible -9 items 20 minutes SR. Limitation-designed for use during school year not too serious & appropriate for classroom administration. Data in last year, although not European. 5-point scale floor and ceiling effects.

Youth Risk Behaviour Surveillance Survey (YRBS)	**✔✔✔**	Strong validity and moderate reliability. Track record for population monitoring. Simple, reasonable validity and reliability and been widely used. Excellent method. Multiple assessments of measurement properties.	Used in large scale population monitoring. Tested for large age range (10-21). Inclusion of sedentary questions is a bonus. 5 items should make time to self administer not burdensome. Doesn't require recall of minutes so less variability in estimates. Not adaptable if criterion of 60 minutes of activity changes in future.

Health Behaviour in School Aged Children (HBSC)	✔	Strong validity and strong reliability. Feasible and widely used. No information about validity in relation to an objective measure of activity - fitness only.	Used in large scale population monitoring in Europe and elsewhere. Tested for large age range of teenagers (11-16). Inclusion of sedentary questions is a bonus. 2 items should make self administration time short. Not adaptable if criterion of 60 minutes of activity changes in future.

Children's Leisure Activities Study Survey (CLASS)	✔	Moderate validity weak reliability. No information about validity in relation to an objective measure. (Parental report unlikely to be better than kids report so not clear why criterion).	Requires more resources as is interviewer administered. Low reliability puts it in the lowest category. Easy to understand.

Teen Health Survey	**✔✔✔✔**	Reasonable validity and moderate reliability. reliability is over a 9 week period, which is much longer than typical and may have reduced test-retest correlations compared to those with a shorter recall period. Produces MVPA. Narrow age range means that validity work needs to be conducted for younger ages. Good brief surveillance measure that is an adaptation of the YRBS survey. Use for younger children to be questioned.	Recall period of last 7 days self report. Short and self report makes it feasible.

School Health Action, Planning and Evaluation System (SHAPES) Physical Activity Questionnaire	**✔✔**	Strong validity and acceptable reliability. Good reliability and validity but too long for surveillance work.	The questionnaire is still well received by schools. Applicable to children about 10 year and older in school setting. Drawback is its use in only one country.

Finnish Twin Cohort Study	✔	Rating reflects the very narrow age range associated with its use. Good reliability and validity, relatively simple to complete. Could be used for longer surveillance measures. Items may be a little hard for younger children to understand. Good method but only for adolescents and only for leisure-time pa and sport. Excellent R and F, good V with no objective pa criterion Single assessment.	Tests of properties limited to only teenagers 16 years and older.

## Results

Of the 20 instruments receiving detailed assessment, 4 had no reported reliability data, and a further 3 instruments had weak reliability (see Table 1). For validity, few, if any, were expected to report strong validity due to the nature of self-report instruments with young people. Only two instruments (SHAPES, Finnish Twin Cohort Study) received a strong rating for validity based on strength of statistical value as set out in Table 2. However, these instruments were seen to have limitations for population surveillance. SHAPES was considered to be too long for population surveillance systems, with 45 items, and the Finnish instrument has only been tested with a limited age range.

Three scales received support, based on judgments from the expert group and the authors. The three instruments selected were the PAQ-C/PAQ-A, YRBS, Teen Health Survey. All of these received support from at least 3 of the 4 experts. No other instrument was supported by more than one expert.

### PAQ-C/PAQ-A (Physical Activity Questionnaire)

There are two versions of this scale, with one for children (C) aged 8-14 y [20], and one for adolescents (A) aged 14-20 y [21]. For example, the PAQ-C requests responses for the last 7 days by asking participants to check a list of activities for frequency of participation on a scale from 'no', 1-2 times per week, 3-4, 5-6, to 7 times or more. Questions are also asked about physical activity in PE lessons, recess, at lunch time, right after school, and evenings, as well as 'the last weekend'. A measure of frequency of participation is requested for each day, ranging from 'none' to 'very often'.

The PAQ-C is a self-administered 7-day recall questionnaire. It is intended to measure habitual moderate-to-vigorous physical activity in children and was developed for use in the Saskatchewan Pediatric Bone Mineral Accrual Study [18]. The PAQ-A is a slightly modified version for adolescents. The instruments were produced using appropriate development methods, moreover they are fairly short and easy to use, thus were viewed as suitable for use in surveillance and monitoring. Validity data exist, using objective assessment of physical activity. However, the PAQ is designed for assessment only during the school year, and there are no data showing use in Europe.

### YRBS (Youth Risk Behaviour Surveillance Survey)

The YRBS was developed in the early 1990s and has been extensively used since, including for large scale surveillance across a good range of children and youth in the USA [22,23] (see http://www.cdc.gov/HealthyYouth/yrbs/index.htm). Respondents can report physical activity for the past year or past week, and it comprises only five items for both moderate and vigorous physical activity. A measure of sedentary behaviour is also included, and this may prove useful as the focus on sedentary behaviour increases [24]. Validity data was good for this instrument, including convergent validity with accelerometry.

### The Teen Health Survey

This is a 2-item instrument developed for adolescents. Items were shortened and adapted from the YRBS and tested alongside other instruments for possible use in primary health care [25]. Items also formed part of the CITY100 (Correlates of Indoor Tanning in Youth) survey [26]. The recall period is either the previous 7 days or typical week, and data exist showing moderate-to-good associations with accelerometry. Its applicability to younger children (i.e., pre-teens) has not been tested and may be questionable.

Other instruments that received some support from the expert group did so usually for reasons of ease of administration. These included the APARQ (but has weak validity), the instrument used in the large HBSC study (has limited validity against objective measures of physical activity), CLASS (has weak reliability), and, as stated earlier, SHAPES (meets many of the criteria but has too many items for surveillance), and the Finnish Twin Cohort Study (limited age range).

## Discussion and Conclusions

A robust and useful measure of physical activity is crucial to good population surveillance which, in turn, is a central component of a comprehensive public health response aimed at increasing levels of participation. There is significant interest across Europe to advance both the quality and the comparability of measures of activity in adults and young people [27]. In recent years much work has been undertaken to develop a suitable, internationally recognised, tools for population surveillance and measurement in adult populations [28,29]. However, for young people, assessment using self-report instruments remains particularly problematic for the reasons stated previously and less work has been undertaken to identify and, if needed, develop suitable tools. In young people, measurement error is of a particular concern due to issues of recall. In addition, instruments are only likely to pick up types of physical activity that can easily be recalled and are therefore 'retrievable' from memory. This may miss some of the short and sporadic bursts of activity common for younger children.

Objective methods for the assessment of physical activity are now more common and more feasible, largely because both the cost and complexity have been addressed. This has resulted in trials of their use assessing large samples of young people [30,31], nonetheless it is likely that self report instruments will be required for some time yet, if mainly for reasons of cost. Even if accelerometers become the instrument of choice, self-report instruments will continue to be required as information on both the type and context of physical activity is also important. Moreover, the design of effective interventions requires an understanding of what physical activity people do alongside how much they do. The PAQ scales may be best placed for this as far as the selected scales are concerned.

For use in population level surveillance systems and tracking trends over time, physical activity measures need to demonstrate not only validity and reliability but also ease of administration, particularly if administered in a larger survey assessing other health behaviours. These all present challenges and, as such, there is no existing instrument that easily satisfies all criteria. That said, following a detailed evaluation, three instruments were identified as potentially most suitable for use: PAQ-C/PAQ-A, YRBS, Teen Health Survey (itself a shortened modification of the YRBS). Those undertaking population surveillance of youth physical activity are recommended to consider these instruments and to evaluate their appropriateness for the type of assessment that may be required. While population surveillance of youth physical activity is strongly recommended, it is evident from this review that further research is needed to establish suitable measures and demonstrate effective use in national surveillance systems aimed at young people. Such work should be encouraged particularly where is can also combine with an objective assessment.

## Abbreviations

APARQ: Adolescent Physical Activity Recall Questionnaire; CLASS: Children's Leisure Activities Study Survey; MVPA: Moderate to Vigorous Physical Activity; PAQ: Physical Activity Questionnaire; SHAPES: School Health Action Planning and Evaluation System; USA: United States of America.

## Competing interests

The authors declare that they have no competing interests.

## Authors' contributions

SJHB led the day-to-day management of the project, carried out some of the analyses and led the writing of the manuscript. TG was closely involved in the day-to-day management of the project, carried out some of the analyses and contributed to the writing of the manuscript. NP conducted some of the analyses and contributed to the writing of the manuscript. FCB was the PI for the grant, led initial project planning, reviewed data analysis, and contributed to the writing of the manuscript. All authors read and approved the final manuscript.

## Supplementary Material

Additional file 1 **Table S1: Table of activity-based instruments selected for detailed assessment**. Detail on each selected instrument. Click here for file
