# Supplementary material for: An assessment of self-reported physical activity instruments in young people for population surveillance: Project ALPHA
Source: Int J Behav Nutr Phys Act. 2011 Jan 2;8:1. doi: 10.1186/1479-5868-8-1 (PMC3019119; doi:10.1186/1479-5868-8-1)
Supplement: Additional file 1 — Table S1: Table of activity-based instruments selected for detailed assessment. Detail on each selected instrument. [file 1479-5868-8-1-S1.DOCX]

**Supplementary Table**. Table of activity-based instruments^[[1]](#footnote-1)^ selected for detailed assessment^[[2]](#footnote-2)^

| **Instrument** | **Indicative References** | **Details of PA assessment^[[3]](#footnote-3)^** | **Age Range**  **&**  **Indicative Countries** | **Reliability** | **Validity** | **Comment** |
| --- | --- | --- | --- | --- | --- | --- |
| SAPAC - Self-Administered Physical Activity Checklist | Sallis, et al., (1996) Validation of interviewer- and self-administered physical activity checklists for fifth grade students, *Med Sci Sports Exerc. 28*, 840-851.  McMurray, et al., (2004). Comparison of two approaches to structured physical activity surveys for adolescents, *Med Sci Sports Exerc. 36,* 2135-2143.  *– used a modified SAPAC (more activities and in a 3-day format)* | 1. previous day PA (performed for more than 5 minutes at a time)  2. 1 day  3. self-report checklist  4. 21 activities with space for 4 ‘other’  5. # activities, total minutes MVPA, PA MET score, weighted activity MET score  6. asks for duration and intensity | Sallis et al., (1996) Fifth grade; mean age =12.8y + 0.88  USA  McMurray et al., (2004) mean age 12.5y + 1.1  USA | Sallis et al., (1996).  ICC with PACI (N=120). r=0.64-0.79  Reported 25% more MVPA on SAPAC than PACI  McMurray et al., (2004). 7 day test – retest correlation for MVPA r=0.667 (n=66); girls r=0.713 (n=47); boys r=0.673 (n=19). 7 day test- retest correlation for VPA r=0.627 (n=66); girls r=0.642 (n=47); boys r=0.811 (n=19) | Sallis et al., (1996). **Convergent validity against HR data: (**N= 115) r=0.28 heart rate #activities; r=0.58 MVPA mins; r=0.60 MVPA METS; r=0.59 Weighted MVPA METS  Sallis et al., (1996).  **Convergent validity against caltrac accelerometer:** (N=119) r=0.02 caltrac #activities; r=0.30 MVPA mins; r=0.32 MVPA METS; r=0.32 Weighted MVPA METS  McMurray et al., (2004). **Convergent validity against actigraph accelerometer: MVPA counts** r=0.239 (n=107); girls r=0.265 (n=75); boys r=0.340 (n=32); **VPA counts** r=0.281 (n=107); girls r=0.312 (n=75); boys r=0.289 (n=32) | Modification and extension of Sallis et al RQES, 64:25-31, 1993  Also has interview format (PACI). Sallis et al., (1996)  Data do not strongly favour SAPAC over PACI or vice versa  Appears in modified forms (usually changes to list of activities provided). Generally retains similar psychometric properties  Recall period has been varied up to two weeks  Used in several high profile studies (e.g., CATCH) |
| Adolescent Physical Activity Recall Questionnaire  (A-PARQ) | Booth et al., (2002). The reliability and validity of the adolescent physical activity recall questionnaire. *Med Sci Sports Exerc, 34,* 1986-1995*.*  Booth, et al., (2005). Methods of the NSW schools physical activity and nutrition survey (SPANS). *J Sci Med Sport, 8(3),* 284-293.  Li, et al., (2006). Factors associated with adolescents' physical inactivity in Xi'an City, China,' *Med Sci Sports Exerc, 39,* 2075-2085*.* | 1. organised sport, non-organised sport  2. normal week in school year, recalled separately for summer and winter terms.  3. SR  4. 4 grids (activity category by winter/summer)  5. categorical outcome: vig (vig PA 3x week for 20min), mod (3.5hrs MPA/week over 5 sessions), inactive (not in other 2 categories), sometimes vig and mod are combined.  Continuous outcome: EE from METS for organised and non-organised activities.  6. free-response (although common activities are listed as prompts). Frequency and average duration of each activity is recorded. Durations of <10mins are excluded during processing.  Winter and summer recalls are NOT combined. | Booth et al., (2002) 13 & 15y Australia  Li, et al., (2006). 11-17y  China | Booth et al., (2002) N=226, 7 high schools  Test-retest (2 weeks)  Weighted kappa:  3 Category outcome ranged 0.33-0.71 and 0.39-0.71 for summer and winter.  2 Category outcome ranged 0.34-0.74 and 0.25-0.68 for summer and winter.  Continuous outcome:  Total EE range  Summer ICC = .30-.86; Winter ICC = .36-.91  Summer Spearmans rho = .64-.81; Winter Spearmans rho = .52-.74 | Booth et al (2002). N=2026, 44 high schools  **Convergent validity: EE total (organised and non-organised activities) against laps on multi-stage fitness test**: Spearman’s rho=0.147 and 0.208 for grade 8 girls and boys; Spearman’s rho=0.139 and 0.391 for grade 10 girls and boys.  Booth et al. (2005) and Li et al. (2006) cite Booth et al. (2002) when describing the A-PARQ. | 20 mins to complete  Used in SPANS (Schools Physical Activity and Nutrition Survey)  Li et al., (2006) adapted for Chinese adolescents to include popular Chinese activities such as jumping rope and parcel shooting, and activities such as cricket and squash were deleted. |
| Swedish Adolescent Physical Activity Questionnaire (SwAPAQ) | Ekelund., et al (2006) The criterion validity of a last 7-day physical activity questionnaire (SAPAQ) for use in adolescents with a wide variation in body fat: The Stockholm Weight Development Study, *Int J Obes, 39,* 1019-1021.  Questionnaire available for download at  www.mrc-epid.ac.cam.uk  Corder, et al. (2009). Is it possible to assess free-living physical activity and energy expenditure in young people by self-report? *Am J Clin Nutr*, *89*, 1-9. | 1. school, transport & leisure time PA plus sitting time  2. last 7 days  3. self-report  4. 25  5. frequency, duration and intensity used to generate outcomes  6. response format unclear; broad categories offered | Ekelund, et al (2006)  mean age 16.8y + 0.4  Sweden  Corder et al. (2009)  British 16-17y  UK | Not reported | Ekelund et al (2006). **Convergent validity (criterion):** (N=49) Correlations between total volume of self-reported PA (total MET-minutes) and MTI accelerometer assessed variables of PA. Adjusted for gender: against time spent sedentary (min day) r= -0.44; against time spent in PA (min day) r=0.51; against total counts per day r=0.48; against total counts per minute per day r=0.44  Corder et al (2009) **Convergent validity (criterion):** (N=24)  Spearman rho:  SwAPAQ MVPA v accelerometer MVPA = 0.23; SwAPAQ PAEE v DLW-EE = 0.40. |  |
| Physical Activity Questionnaire for Older Children (PAQ-C)  Physical Activity Questionnaire for Adolescents (PAQ-A) | Crocker, et al., (1997). Measuring general levels of physical activity: Preliminary evidence for the Physical Activity Questionnaire for Older Children. *Med Sci Sports Exerc, 29,* 1344-1349*.*  Crocker, et al., (2000) Children's physical activity and physical self-perceptions. *J Sports Sci, 18,* 383-394*.*  Kowalski, et al., (1997a). Validation of the Physical Activity Questionnaire for Older Children. *Pediatr Exerc Sci, 9,* 174-186*.*  Kowalski, et al., (1997b). Convergent validity of the Physical Activity Questionnaire for Adolescents. *Pediatr Exerc Sci, 9,* 342-352*.*  Janz, et al., (2008). Measuring Activity in Children and Adolescents Using Self-Report: PAQ-C and PAQ-A. *Med Sci Sports Exerc, 40,* 767-772*.*  Moore., et al. (2007) Validation of the Physical Activity Questionnaire for Older Children in children of different races. *Pediatr Exerc Sci*, 19:6–19. | 1. Moderate to vigorous PA levels during the school year  2. 7-day recall  3. Self-report  4. 9 items  5. Outcome measure: composite PA; LMVPA (longer-than-10mins-MVPA)  6. 5-point rating scale | PAQ-C  8-13y;  PAQ-A:  14-18y  Canada, USA | **PAQ-C**: Crocker et al., (1997). (N=215) Test-retest (1-week): r=0.75 (males) r=0.82 (females)  Internal reliability >0.80.  **PAQ-C & PAQ-A:** Janz et al (2008) (N=210) **Internal consistency;** standardised Cronbach alphas ranged from 0.72 to 0.88. | **PAQ-C**  Kowalski et al., (1997a). (N=89). **Convergent validity**:  Relationships for PAQ-C and activity rating question, a teacher's rating of physical activity, and moderate to vigorous PA assessed by a separate inventory (r=0.45 to r=0.63). **Construct validity:** positive relationship with perceived competence (r=0.48) **Divergent validity:**  no relationship with behavioural conduct (r=0.16).  Kowalski et al., (1997a). (N=97). **Convergent validity:** r=0.39 caltrac; r=0.41-0.43 with other self-reports - PAR interview and leisure time exercise questionnaire; r=0.28 step test (fitness test).  **PAQ-A**  Kowalski et al., (1997b). (N=85) **Convergent validity:** r=0.73 activity rating; r=0.57 Leisure Time Exercise Questionnaire; r=0.33 Caltrac; r=0.59 7-day physical activity recall interview.  Janz et al., (2008). (N=49) **Convergent validity.** Rho=0.47 for total activity and actigraph; rho=0.49 for MVPA and actigraph. Rho improved to.56 and .63 when questions about PA and lunch activity were removed. | PAQ-A is the same as PAQ-C but doesn’t include a question about morning recess  Both designed to be used during the school year and not during vacation periods |
| Youth Risk Behaviour Survey (YRBS) | Brener, et al., (2002). Reliability of the 1999 Youth Risk Behaviour Survey Questionnaire. *J Adolesc Health, 31*, 336-342.  Brener, et al., (1995). Reliability of the Youth Risk Behaviour Survey Questionnaire, *Am J Epi, 141*, 575-580.  Troped, et al., (2007). Reliability and validity of YRBS Physical Activity Items among Middle School Students,' *Med Sci Sports Exerc, 39,* 416-425.  Survey available: www.drjamessallis.sdsu.edu/measures/html | 1. school and leisure time PA; vigorous, moderate, strength exercises, sport  2. past week and past year  3. self-report  4. 5 PA items  5. Physical activity behaviours defined as: watch 2 hours or more of television on an average school day; attend physical education class 1 or more days a week; exercise more than 20 minutes during physical education class; played on 1 or more sports team during the past 12 months; injured during physical activity 1 or more times during the past 12 months  6. frequency of participation | Brener et al., 2002.  mixed race high school students 13-18y.  Brener et al. 1995. grades 7-12.  Troped et al. 2007. 12.7y +0.6  USA, Philippines, Canada | Brener et al., (2002). (N=4619)  PA behaviours assessed, on average 15 days apart.  **Kappa statistics:** 41.1-84.8%. Kappas did not differ by gender, grade, race/ethnicity.  Brener et al. (1995) (N=1679) PA behaviours assessed, 2-weeks apart  **Kappa statistics:** 64.2-91.1%  Troped et al (2007). Moderate and vigorous physical activity assessed by YRBS between 1-40 days apart  **Test-retest ICC** for moderate physical activity items: 0.51 (0.59 for girls and 0.37 for boys)  ICC for vigorous physical activity items: 0.46 (0.47 for girls and 0.42 for boys). | Troped et al (2007).  **Convergent validity.** (N=128)  Concordance between YRBS and Actigraph moderate physical activity measures was highest using accumulated accelerometer minutes. Sensitivity of the moderate YRBS item ranged from 0.19 to 0.23 for four comparisons, and specificity was 0.74-0.92.  For vigorous activity: sensitivity was high (0.75-0.92) compared with the four actigraph measures. Specificity was low (0.23-0.26) | ‘Physical activity’ items include measures of sedentary behaviour and injury. |
| Health Behaviour in School Aged Children (HBSC) | Booth, et al., (2001). The reliability and validity of the physical activity questions in the WHO health behaviour in schoolchildren (HBSC) survey: a population study, *Brit J Sports Med, 35,*263-267  Samdal, et al., (2006). Trends in vigorous physical activity and TV watching of adolescents from 1986 to 2002 in seven European Countries, *Eur J Pub Health, 17,* 242-248*.*  Janssen, et al., (2005). Comparison of overweight and obesity prevalence in school-aged youth from 34 countries and their relationships with physical activity and dietary patterns. *Obes Rev, 6,* 123-132 | 1. out-of-school moderate-to-vigorous PA (“.. out of breath or sweat”)  2. “usually”  3. self-report  4. 2 PA items; 2 sedentary behaviour items  5. frequency and duration  6. tick boxes | 11-16y  >30 countries in network (mainly European).  Scale established in 1983/84 in  Austria, England, Finland, Norway. 35 countries from 2001/02. | Booth et al (2001). N=121 year 8 students and N=105 year 10 students.  2-week test-retest: **Kappa statistics:** 67-85%  Samdal et al (2006) cite an unpublished report by Torsheim et al 1995 - Intraclass correlation coefficient (ICC) = 0.74 | Booth et al (2001).  N=1072 year 8 students and N=954 year 10 students **Convergent validity:**  Higher fitness scores for those with higher self-reported PA. |  |
| Children's Leisure Activities Study Survey (CLASS) | Telford, et al., (2004). Reliability & validity of physical activity questionnaire for children: The Children's Leisure Activities Study Survey, *Ped Exerc Sci, 16,* 64-78. | 1. physical activity  2. ‘typical week’  3. self-report (SR) for 10-12y olds; parental proxy (PP) for 5-6y and 10-12y olds  4. checklist of activities  5. frequency and duration  6. tick boxes | 5-6 y, 10-12y  Australia | Telford et al (2004): (n=56, 5-6 year olds and their parents; n=111 10-12 year olds and their parents)  2-week test-retest reliability (% agreement) for individual activities = 62-94% (SR & PP); judged ‘moderate to substantial’.  ICC for SR & PP varied for different intensity levels, parental education, and child gender (ICC = 0.15 to 0.89). | Telford et al (2004).  **Convergent validity:** Individual item agreement between SR and PP was good. Moderate, vigorous and total PA scores were lowly correlated.  Telford et al (2004).  **Criterion-related validity:**  Low correlations between CLASS and accelerometer scores for both SR and PP instruments for both age groups.  Telford et al (2005). **Criterion-related validity:**  Zero-to-low correlations between PP measure and accelerometers. |  |
| Godin Leisure-Time Exercise Questionnaire (LTEQ) | Godin & Shephard, (1985). A simple method to assess exercise behaviour in the community. *Canadian J Appl Sport Sci, 10,* 141-146.  Sallis, et al., (1993). Seven-day recall and other physical activity self-reports in children and adolescents, *Med Sci Sports Exerc, 25,* 99-108.  Fulkerson, et al., (2004). Depressive symptoms and adolescent eating and health behaviours: a multifaceted view in a population-based sample, *Prev Med, 38,*  865-875.  Gao, et al., (2006). Reliability and Validity of a brief tool to measure children's physical activity, *J Physical Activity Health, 3,* 415-422  Scerpella, et al., (2002) Validation of the Godin-Shephard questionnaire in prepubertal girls. *Med Sci Sports Exerc, 34,* 845-850*.* | 1. strenuous, moderate and mild exercise for more than 15mins  2. “A 7-day period”  3. self-report  4. 4 items  5. Strenuous score x 9, moderate x 5, light x 3. “Weekly leisure activity” is product of above. “Total leisure activity” includes frequency (e.g., strenuous = 9 x frequency).  6. Write in frequency estimates | From 9y  Canada, UK, USA | Sallis et al., (1993)  Test-retest (2-wk) of total score: 11y r=0.69, 13/14y r=0.80, 16y r=0.96.  Gao et al., (2006).  Test-retest (1 wk):  Ages 12-14y (N=250) r=0.48-0.68 | Gao et al., (2006).  **Criterion-related validity (N=114):** Number of days per week of participation by LTEQ v. accelerometer data: strenuous r=0.23; moderate r=0.13.  Scerpella et al., (2002). **Criterion-related validity:** Relationship between total LTEQ score and other activity measures in 7-11y girls (N=61):  Activity Rating Scale (PACI) r=0.25; caltrac accelerometer r=0.10; self-reported organised activity (hrs/wk) r=0.30; combined (above) r=0.38.  Scerpella et al (2002). Based on comparison with multiple single day measures of activity, the LTEQ did not provide a good estimate of habitual activity in preadolescent females |  |
| Seven-Day Physical Activity Recall (7D-PAR) | Sallis, et al., (1993). Seven-day recall and other physical activity self-reports in children and adolescents. *Med Sci Sports Exerc*, 25, 99-108.    Bratteby, et al., (1997). A 7-day activity diary for assessment of daily energy expenditure validated by the doubly labelled water method in adolescents. *Euro J Clin Nutr, 61,* 585-591*.* | 1. very hard and moderate PA for more than 15mins  2. Previous 7 days  3. interview administered  4. N/A  5. METs  6. Recall previous day then work back. | 11-16y  USA  Sweden | Sallis et al., (1993).  Test-retest (9 weeks) for KKD: r=0.47 (grade 5, N=36); r=0.59 (grade 8, N=36); r=0.81 (grade 11, N=30).  For moderate activity r=0.13 (grade 5, N=36); r=0.0.11 (grade 8, N=36); r=0.0.75 (grade 11, N=30). | Sallis et al., (1993).  **Criterion validity**  Relationship between hours of recalled very hard activity and heart rate monitoring time periods >160beats/min r= 0.29 (5th grade), r=0.45 (8th grade), r=0.72 (11th grade) |  |
| Teen Health Survey | Butcher, et al., (2008) Correlates of Physical Activity Guideline Compliance for Adolescents in 100 U.S. cities. *J Adolesc Health, 42,* 360-368  Prochaska et al., 2001 A Physical Activity Screening Measure  for Use With Adolescents in Primary Care. *Arch Pediatr Adolesc Med*, 155, 554-559. | 1. MVPA (≤3 METS) 2. 7-d and typical week 3. self-report 4. 2 5. N/A 6. free response | 14 - 17y  USA | Prochaska et al., 2001 (N=138)  Test-retest from same day to up to one month: ICC controlled for time to retest: r=0.76, ICC = 0.88 for same day test- retest; ICC = 0.53 for one month test-retest | Prochaska et al., 2001 **Criterion Validity**  Survey vs. accelerometer r=0.40. Stronger for boys (N=36): r=0.42 than for girls (N=63): r=0.32. |  |
| Four by One-day Recall Physical Activity Questionnaire | Cale, et al., (1997) The physical activity levels of English adolescent boys. *Physical Education & Sport Pedagogy, 2,* 74-82.  Khunti, et al., (2007) Physical activity and sedentary behaviours of South Asian and white European children in inner city secondary schools in the UK. *Family Practice,* doi:10.1093/fampra/cmm013*.* | 1. PA at school, sport at school, leisure time PA, leisure time sport 2. 24-h 3. Self-report 4. 2 forms; weekday and a weekend day. Form split into parts: morning, afternoon and evening 5. PA measured in terms of intensity; MET values assigned to activities 6. Tick boxes | 11-18y  UK | Cale et al., (1997).  4-week test-retest: (N=12), 11-14y r=0.62 | Cale et al., (1997).  **Criterion validity**  (N=20) Survey vs. HR monitor r=0.61; vs.  observational method r=0.79. |  |
| Computerised Activity Recall (CAR) | McMurray et al., (1998) Comparison of a computerised physical activity recall with a triaxial motion sensor in middle-school youth. *Med Sci Sports Exerc, 30, 1238-1245.* | 1. All PA and sedentary behaviour of at least 15m duration  2. Previous day  3. Self-report (via computer)  4. Menu driven behaviour categories of over 200 behaviours  5. Time of day data also collected  6. Duration of behaviour | grades 6-8  USA | McMurray et al (1998). (N=22) grade 6-8 students.  1-2 week test- re-test ICC for total EE= 0.947. | McMurray et al (1998).  **Criterion validity**  CAR vs. Tritrac for total EE r=0.510. Kappa statistic = 0.398 indicating a low level of agreement between methods. |  |
| Children’s Physical Activity Interview | Simons-Morton, et al., (1997) Physical activity in a multiethnic population of third graders in four states. *Am J Pub Health, 87,* 45-50.  Simons-Morton, et al., (1994). Validity of the Physical Activity Interview with preadolescent children. *Research Quarterly for Exercise and Sport, 65*(1), 84-88. | 1. All PA during waking hours 2. Previous day recall 3. Interview 4. Interview segmented into 5 time slots 5. Filled in a Physical Activity Record prior to interview 6. Free response directed by the interviewer | Grades 3 & 5  USA |  | Simons-Morton et al., (1994).  Pearson correlations between reported activity minutes and monitored minutes of high intensity activity (180% of resting HR):  3rd Grade (N=27) r=0.57; 5th Grade (N=21) r=0.72 |  |
| Cardiovascular Risk in Young Finns | Telama, et al., (2000) Decline of physical activity from youth to young adulthood in Finland. *Med Sci Sports Exerc, 32,* 1617-1622*.*  Telama, et al., (2005) Physical Activity from Childhood to Adulthood: A 21-year tracking study. *Am J Prev Med, 28,* 267-273.  Yang, et al., (2007) Testing a model of physical activity and obesity tracking from youth to adulthood: the cardiovascular risk in young Finns study. *Int J Obesity, 31,* 521-527*.* | 1. Leisure time PA, sports club/competitions 2. Habitual activity (PA Index: PAI) 3. Self-report 4. 5 items 5. Frequency and intensity 6. free response | 3, 6, 9, 12, 15 & 18y  Finland | Telama et al ., (2000). Internal consistency- Cronbach alpha: 0.44 – 0.76 | Telama et al., (2005).  **Criterion-related validity:** PAI scores correlated with measures of fitness: r = 0.2 – 0.53. |  |
| ACTIVITY video questionnaire | Tremblay, et al., (2001) Preliminary evaluation of a video questionnaire to assess activity levels of children. *Med Sci Sports Exerc, 33,* 2139-2144 | 1. School-day PA 2. 1-d recall 3. Self-report 4. 10 items 5. n/a 6. Checklists | 3rd grade  Canada |  | Tremblay et al (2001).  **Criterion-related validity:** Pearson correlations with ACTIVITY score:  Accelerometer r=0.40,  Heart rate r=0.50, |  |
| Youth Media Campaign Longitudinal Survey  (YMCLS) | Welk et al., (2007). Reliability and validity of questions on the Youth Media Campaign Longitudinal Survey. *Med Sci Sports Exerc, 39,* 612-621*.*  Heitzler et al., (2006). Correlates of physical activity in a national sample of children aged 9 to 13 years. *Prev Med, 42,* 253-260 | 1. Out-of-school activities, leisure time PA 2. 7-d recall and 1-d recall 3. Structured phone interview 4. ? 5. n/a 6. Free response | 9 -13y  USA | Welk et al (2007).  Test-Retest:  (1 week)  ICC=  0.78 (organised activity) 0.60 (free-time activity) 0.60 (total weekly activity). | Welk et al (2007) . **Criterion-related validity:**  Total weekly activity and accelerometer (r=0.24), activity log (r=0.46).  Activity time and sessions on previous day with accelerometer (r=0.53 and 0.37) and the activity log (r=0.37 and 0.47). Correlations between the YMCLS and the activity log were higher for organised activity (r=0.72) than for free-time activity (r=0.46). | Reliability and validity coefficients were similar for boys and girls, but older youth (11-13 years) had higher coefficients than younger students (9-10 years) |
| School Health Action, Planning and Evaluation System (SHAPES) Physical Activity Questionnaire | Wong et al., (2006) Reliability and validity of a school-based physical activity questionnaire. *Med Sci Sports Exerc, 38,* 1593-1600. | 1. All PA 2. 7-d recall 3. Self-report 4. 45 items (multiple choice) 5. Responses are provided by indicating the number of hours and 15-min increments that each type of PA was performed for each day of the previous week. 6. Tick boxes/multiple choice | grades 6 – 12  Canada | Wong et al (2006). (N=2812)  1 week test-retest:  Kappa/weighted kappa coefficient = 0.57±0.24 | Wong et al (2006).  **Criterion-related validity:** (N=67)  Spearman’s correlation with MTI accelerometer r=0.44. |  |
| Finnish Twin Cohort Study | Aarnio, et al., (2002a) Stability of leisure-time physical activity during adolescence - a longitudinal study among 16-, 17- and 18-year old Finnish youth. *Scan J Med Sci Sports, 12,* 179-185*.*  Aarnio, et al., (2002b) Associations of health related behaviour, social relationships, and health status with persistent physical activity and inactivity: a study of Finnish adolescent twins. *Brit J Sports Med, 36,*360-364.  Aarnio, et al., (1997)  Associations of health related behaviour, social relationships, and health status to physical activity among 16 year old boys and girls. *Scand J Soc Med, 3*, 156-167. | Aarnio, et al., (2002a)   1. Leisure time PA; frequency of leisure time PA outside of school, perception of physical fitness and intensity of PA 2. Habitual PA 3. self-report 4. 2 items 6. Scale (1-6)   Aarnio, et al., (2002b)   1. Sport 2. Habitual sporting activity 3. self-report 4. 19 items 5. Report any sport they had participated in; groups were then divided into ‘aerobic’, ‘power’ and ‘other’, also organised and non-organised sport was assessed 6. Check list | 16 - 18y  Finland | Aarnio et al (1997).  Test-Retest: (1 month):  Self-reported fitness:  Boys: r= 0.76  Girls: r=0.80  Self-reported Intensity:  Boys: r=0.79  Girls: r=0.75 | Aarnio et al (2002a).  **Criterion-related validity:** (N=32) questionnaire-reported fitness and VO2max rho= 0.47, interview-based intense physical activity and VO2max rho = 0.61, questionnaire-reported frequency of activity and VO2max rho=0.45.  The correlation of interview-based intense physical activity with questionnaire fitness was r=0.62 and with questionnaire frequency r=0.52. |  |
| Physical Activity and Lifestyle Questionnaire  (PALQ) | Argiropoulou et al., (2004) Validity and reliability of physical activity measures in Greek high school age children. *J Sports Sci Med, 3,* 147-159. | 1. Leisure time PA 2. 7-day and habitual 3. Self-report 4. 27 activities 6. Combination of free response and tick boxes | Mean age 13.73y (SD 0.8)  Greece | Argiropoulou et al (2004). (N=40)  PALQ 2 week test-retest ICC=0.52 | Argiropoulou et al (2004).  **Criterion-related validity:** (N=40) accelerometer and PALQ r=0.53 |  |
| Physical Activity and Exercise Questionnaire (PAEQ) | Schmidt et al., (1998). The Singapore Youth Coronary Risk and Physical Activity Study. *Med Sci Sports Exerc, 30*(1), 105-113.  Chia, et al., (2002). Relationships between hours of computer use, physical activity and physical fitness among children and adolescents. *Euro J* *Physical Educ, 7,* 136-155 | 1. Usual PA 2. Habitual 3. Self-report 4. Choose 1 from 4 categories 5. PA described in terms of intensity; very light, light PA, moderate PA & VPA 6. Tick boxes | 6-18y  Singapore |  | Schmidt et al., (1998). **Criterion-related validity:** (N=24) PAEQ v heart rate r=0.67.  **Construct validity:** (N=745) PAEQ v total cholesterol r=0.35, high density lipoprotein r=-0.38, and triglycerides r=-.027 |  |

1. Activity – based instruments are structured around a list of activities with minimal reference to time of day. These contrast with time-based instruments where the day is divided into time blocks and respondents provide dominant activity/energy expenditure for each time block. [↑](#footnote-ref-1)
2. Instrument had to contain some assessment of validity or reliability. [↑](#footnote-ref-2)
3. 1. Physical activity dimension; 2. Recall period; 3. Method and structure of instrument; 4. Number of items; 5. Other relevant information; 6. Measurement format. [↑](#footnote-ref-3)
